# Water pressure effect and influence mechanism of coal mechanical property degradation under mine water immersion conditions

**DOI:** 10.1371/journal.pone.0328477

**Published:** 2025-12-12

**Authors:** Xiaohu Zhu, Zeliang Liang, Jia Tan, Yi Wang, Heng Zhang

**Affiliations:** 1 Xinjiang Yaxin Coalbed Methane Investment and Development (Group) Co., Ltd, Urumqi, China; 2 Xinjiang Yaxin Coalbed Methane Resources Technology Research Co., Ltd, Urumqi, China; 3 Xinjiang Key Laboratory of Coalbed Methane Exploration and Development, Urumqi, China; IGDTUW: Indira Gandhi Delhi Technical University for Women, INDIA

## Abstract

This study investigates the hydro-pressure effects and degradation mechanisms of coal under mine water immersion using a self-developed pressurized water soaking system. Uniaxial compression tests were conducted on water-saturated coal samples to analyze their mechanical behavior under varying water pressures. The results demonstrate that both the strength and elastic modulus of coal samples decrease with increasing immersion pressure. During loading, multiple deformation localization zones develop, and the elastic energy stored in the load-bearing structure diminishes as water pressure rises, reducing its energy storage capacity. Therefore, in the process of plastic deformation, the externally applied energy contributes more to the macroscopic and microscopic damage. When the material is immersed in high-pressure water, its failure mode will shift from tensile dominant to tensile shear composite mechanism, which not only exacerbates internal damage but also significantly accelerates the deterioration process of the fracture surface. Quantitative analysis shows that both surface porosity and probability entropy are positively correlated with immersion pressure. Furthermore, increasing water pressure enhances the pressure-driven water-coal interaction, particularly in acidic mine water environments, leading to the softening, argillization, and dissolution of hydrophilic clay minerals, thereby reducing their relative content. These findings provide theoretical insights for optimizing coal mine hydraulic technologies, enhancing gas extraction efficiency, and ensuring underground stability.

## 1. Introduction

China’s energy landscape is characterized by an abundance of coal but limited oil and natural gas resources, a distribution often summarized as ‘rich in coal, poor in oil, and scarce in gas’ [1–7]. As a result, coal has long served as the dominant and irreplaceable energy source in China and is expected to remain the primary energy contributor for decades to come, with production volumes likely to remain high [8–13]. However, most coal seams in China exhibit complex geological conditions, including soft coal masses, low permeability, high gas content, and significant in-situ stresses, which collectively contribute to frequent coal and gas outbursts. To enhance coal seam permeability, various hydraulic measures—such as coal seam water injection and its derivative techniques (e.g., hydraulic fracturing, hydraulic slotting, and hydraulic flushing)—are widely employed [14–18]. These methods improve gas drainage efficiency and mitigate outburst risks. When water infiltrates the coal’s pores and fractures, it increases moisture content, leading to mechanical weakening [19–24]. This degradation facilitates pressure relief within the coal mass and promotes gas desorption and migration, thereby achieving effective pressure release and gas control.

Currently, the majority of researchers have conducted laboratory-scale investigations into the mechanical degradation characteristics and mechanisms of coal under water immersion, providing theoretical support for the rationality of coal seam hydraulic measures. For instance: Liu et al. [25] analyzed the fractal characteristics and pore structure evolution of coal samples subjected to dry-wet cycles, establishing quantitative relationships between mechanical parameters and structural morphology. Zhong et al. [5] performed cyclic loading-unloading tests on water-saturated coal samples under varying loading rates, amplitudes, and cycle numbers, investigating their strength, plastic hysteresis loops, and acoustic emission (AE) characteristics to elucidate the influence of moisture content on mechanical behavior. Liu et al. [26] employed a Split Hopkinson Pressure Bar (SHPB) system to assess the mechanical degradation of coal samples under different moisture contents. Yao et al. [27] conducted uniaxial compression tests on coal samples with varying water contents, proposing a novel relationship between tensile/shear cracks and failure modes while considering water’s effect on fracture mechanisms. Yao et al. [28,29] demonstrated that water immersion significantly reduces compressive strength and elastic modulus through mechanical testing. Their nondestructive water absorption experiments revealed dynamic changes in coal’s water uptake capacity. Additionally, uniaxial compression and shear tests under varying saturation levels established functional relationships between strength, mechanical parameters, and water content, complemented by AE signal analysis to characterize deformation and failure patterns. Lai et al. [30,31] performed uniaxial compression tests on coal samples under different hydration states, multiscale analyses of hydro-mechanical coupling effects on macro/micro damage evolution, and energy release mechanisms. Their findings indicate that water absorption slows damage progression by absorbing elastic strain energy, leading to increased elastic energy proportion during yielding and reduced dissipated energy, thereby weakening coal’s burst propensity. Wang et al. [32] utilized nuclear magnetic resonance (NMR) to study T2 spectra, pore-throat structures, porosity changes, and image evolution in coal samples under unilateral cyclic water immersion. Their work delineated pore structure evolution and uniaxial compression failure patterns under increasing immersion cycles, revealing water-induced damage mechanisms. Han et al. [33] identified progressive failure characteristics in long-term immersed coal samples, correlating mechanical parameters with saturation and immersion duration while analyzing macroscopic crack propagation behavior. Jin et al [34] studied the relationship between different soaking times and the water absorption rate of coal particles, analyzed the material content of coal particles after soaking, and characterized the water absorption rate of coal particles through X-ray diffraction (XRD), water absorption rate calculation, and water absorption rate measurement. Lyu et al [35] analyzed the compressive strength and deformation damage characteristics of coal samples, revealing the macroscopic and microscopic degradation mechanisms of coal under the coupling of water and gas.

The aforementioned studies have significantly contributed to understanding the deterioration characteristics and mechanisms of coal-rock mechanical properties under water immersion. However, most existing research has focused solely on the influence of factors such as moisture content or immersion duration on coal-rock or coal mass, while neglecting the critical role of pressurized water injection—a key technique in coal seam hydraulic fracturing. Given that coal-rock is typically subjected to pressurized water immersion in practical applications, and the water used often consists of mine water or groundwater, it is imperative to incorporate the hydraulic pressure effect under mine water immersion conditions when investigating the deterioration of coal-rock mechanical properties. To address this gap, the present study builds upon previous research by first utilizing a self-developed pressurized water immersion testing apparatus to treat coal samples under varying hydraulic pressures (0, 2, and 4 MPa) for a consistent immersion duration of 10 days. Subsequently, uniaxial compression tests were conducted using a digital speckle dynamic deformation measurement system to analyze the strength characteristics, deformation and failure behavior, and energy evolution patterns of coal samples under pressurized water immersion. Furthermore, scanning electron microscopy (SEM) and X-ray diffraction (XRD) were employed to examine the microstructural features of fracture surfaces and variations in mineral composition. Finally, the hydraulic pressure effect and its underlying mechanism on the mechanical degradation of coal samples were elucidated. The findings not only provide a theoretical foundation for coal seam hydraulic fracturing technologies but also contribute to enhanced gas extraction efficiency and improved mine safety, thereby offering substantial implications for ensuring safe coal mining operations [36].

## 2. Test program design

### 2.1. Sample preparation

**Fig 1:**
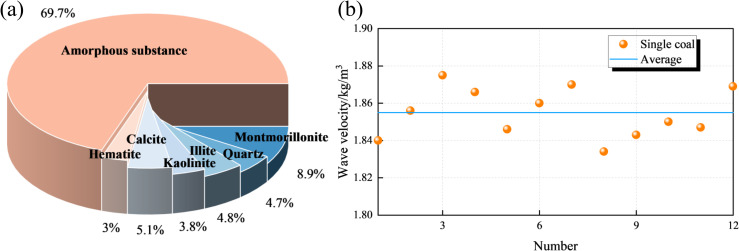
Coal sample sampling location and detection. (a)XRD result. (b)Wave velocity.

The coal samples used in this experiment were collected from a mine in the Baicheng mining area of Xinjiang. Mineral composition analysis conducted via X-ray diffraction (XRD) revealed that the primary mineral constituents included montmorillonite (8.9%), quartz (4.7%), kaolinite (3.8%), calcite (5.1%), illite (4.8%), and hematite (3.0%), as illustrated in Fig 1. The mine water utilized in the tests was obtained from in-situ groundwater seepage within the mine shaft. Chemical analysis indicated that the water sample exhibited weak acidity, with a pH of 6.15, and contained multiple ionic species, including cations such as K⁺(20.42 mg/L), Na⁺(596.55 mg/L), Ca²⁺(872.19 mg/L), Fe³⁺(<0.1 mg/L), Fe²⁺(<0.1 mg/L), and Al³⁺(750.36 mg/L), as well as anions such as Cl⁻(1687.23 mg/L), SO₄²⁻(1622.27 mg/L), CO₃²⁻(123.15 mg/L), and NO₃⁻(1.93 mg/L) (Fig 1a).

To minimize the influence of sample heterogeneity on experimental results, all coal specimens were extracted from the same coal block. Following the coal and rock sampling methodology outlined by the China Coal Research Institute [37], cylindrical coal samples with a diameter of 50 mm were first obtained using a core drilling machine, after which a rock saw was employed to cut them into standard specimens with a height of 100 mm. Prior to testing, preliminary screening was conducted through ultrasonic wave velocity measurements, mass determination, and dimensional verification to ensure sample uniformity. Twelve specimens exhibiting concentrated wave velocity (1.834–1.875 km/s, mean = 1.853 km/s) and density (mean = 1479.55 kg/m³) distributions were selected for experimentation (Fig 1b), as minimal dispersion in these parameters suggests structural homogeneity at the macroscopic level, thereby enhancing the reliability of subsequent test results. The selected specimens underwent drying in a constant temperature and humidity chamber (60°C for 48 h) to eliminate moisture interference [38–44]. These 12 samples were systematically divided into four groups (labeled A–D), with each group containing three replicates to facilitate comparative analysis under controlled experimental conditions.

### 2.2. Test equipment and test scheme

In order to simulate the pressure water immersion effect of coal sample, the author designed a pressure water immersion device, The pressure water immersion device consists of an soaking room, an interface instrument, a hydraulic gauge, a regulating valve, a pressurization valve, and a computer control system. Place the coal sample in the soaking room, with water input through the inlet and regulating the water flow through a valve. When the water is filled, the interface instrument displays the water level; Adjust the water pressure through the pressure valve and observe the water pressure on the hydraulic gauge. The computer control system adjusts the water pressure in real time and records the pressure changes.

**Fig 2:**
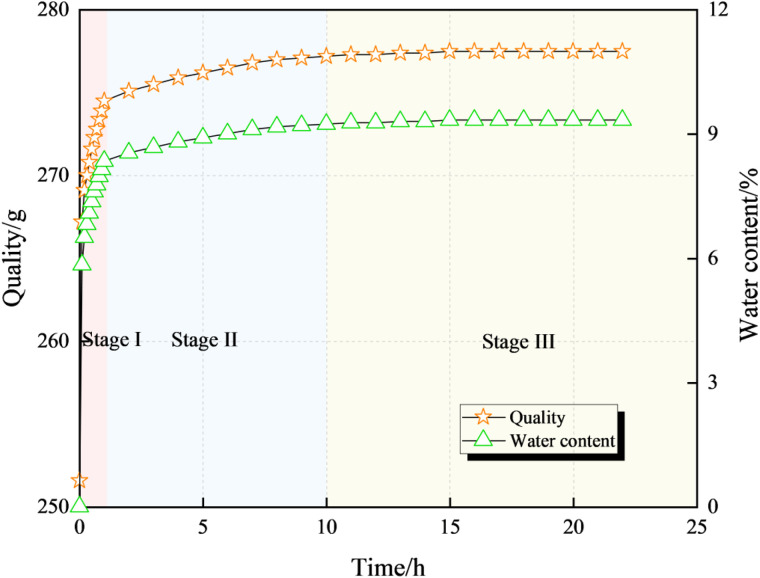
Variation curve of coal sample quality and water saturation rate over time.

Based on the above pressure water immersion device, the saturation treatment was carried out on the coal samples of groups B ~ D first, and group A was not subjected to any treatment: the saturation treatment was carried out with distilled water, and the coal samples were taken out every 10 minutes during the first hour of the treatment to dry the surface water and measure the mass and moisture content, and then taken out every hour for testing, and the curves of the changes of the coal samples’ mass and moisture content with the immersion time were shown in Fig 2, and the curve of the change of the coal samples’ mass and moisture content with immersion time was as shown in Fig 2. When coal samples are subjected to pressurized water immersion, their water content undergoes a three-stage change over time: in the initial 0 ~ 1 hour (Stage Ⅰ), the water content increases rapidly as water quickly infiltrates into the pores and cracks of the coal; during 1 ~ 8 hours (Stage Ⅱ), the water content grows slowly due to the gradual filling of internal defects; after 8 hours (Stage Ⅲ), the water content tends to stabilize, and by 20 hours, the coal samples reach a saturated state where the water content no longer changes significantly. At this time, it can be assumed that the samples have reached a saturated state [45–49], so all of the coal samples of group B ~ D are saturated with water. Therefore, the coal samples of group B ~ D were treated with saturated water for 20 hours. After saturated water treatment, pressure water immersion treatment was carried out for group B ~ D: the pressure water immersion treatment was carried out with mine water, and the immersion pressures of coal samples in group B ~ D were 0, 2 and 4 MPa, respectively, and the immersion length was 10 d. The pressure water immersion treatment was carried out with mine water, and the immersion pressures were 0, 2 and 4 MPa, respectively.

**Fig 3:**
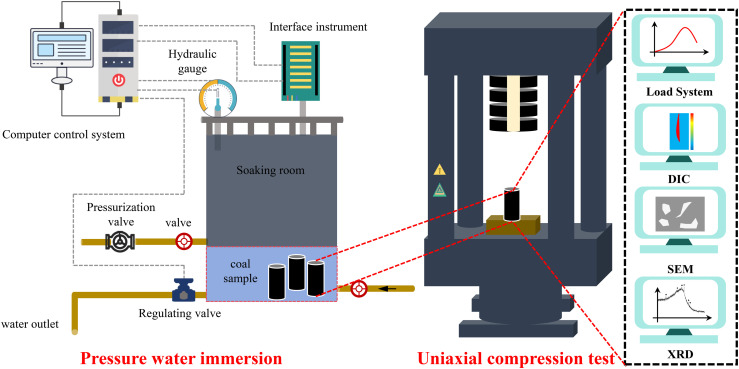
Test equipment and test process.

Uniaxial compression tests were carried out on coal samples from Groups A to D. The main control and monitoring systems for this test included a loading system, a digital interference-coupled dynamic deformation measurement and analysis (DIC) system, a scanning electron microscope (SEM), and an X-ray diffractometer (XRD), and the flow of the test equipment is shown in Fig 3. During the uniaxial compression test, the loading system and DIC were synchronised to ensure the same time parameters for data processing and analysis; after the uniaxial compression test, the SEM was used to observe the rupture fracture morphology of the coal samples, and the mineral components of the coal samples were analysed by XRD.

The loading system is MTS815 rock mechanics testing system, the uniaxial compression test adopts displacement loading control, and the loading rate is 0.005 mm/s; DIC is a digital scattering dynamic deformation measurement and analysis system, which makes scattering field on the surface of coal samples by using artificial paint spraying, firstly, white matte paint is uniformly sprayed on the surface of the coal samples, and then black matte paint is sprayed so that it randomly falls down to the surface of the coal samples to form a black scattering field. Firstly, the white matte paint was evenly sprayed on the surface of the coal sample, then the black matte paint was sprayed so that it randomly dropped to the surface of the coal sample to form a black scattering spot, and finally the black spot scattering field on the surface of the coal sample was produced by the white background.

## 3. Results and analysis

### 3.1. Strength characteristics

**Fig 4:**
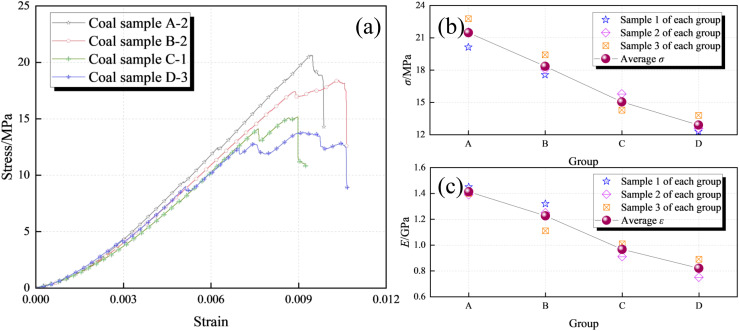
Test results of coal sample mechanical test. (a)Stress-strain curves. (b)strength (σ). (c)Modulus of elasticity (E).

The uniaxial compressive stress-strain curves of the coal samples are shown in Fig 4(a), and the uniaxial compressive strength (σ) and modulus of elasticity (E) of each group of coal samples are shown in Fig 4(b)~4(c). As can be seen from Fig 4(a), the uniaxial compressive stress-strain curves of the coal samples under water immersion at different pressures are basically the same, and all of them experienced four stages of compression density, linear elasticity, plastic yielding and post-peak damage. In the pre-peak stage, the stress-strain curve of Group D coal samples has the longest compression-density stage, the shortest linear-elasticity stage, and the most significant plastic yielding stage; compared with Group D coal samples, the stress-strain curve of Group A coal samples has the shortest compression-density stage, the longest linear-elasticity stage, and the shortest plastic yielding stage; in the post-peak stage, the coal samples were gradually transformed from brittle to ductile damage characteristics with the increase of the water immersion pressure. Comparing with Fig 3(b)~3(c), it can be seen that the average σ and E of the coal samples tend to decrease with the increase of immersion pressure.

This is mainly because as the immersion pressure increases, the water coal interaction between the coal sample and the mineral water strengthens, leading to the precipitation of hydrophilic clay minerals, pore expansion, and a decrease in interparticle cohesion within the coal sample [50–52]. At the same time, this pressure also promotes the propagation and development of macroscopic and microscopic cracks, reduces the difficulty of surface friction of macroscopic cracks, thereby exacerbating internal damage to the coal sample, weakening its solid load-bearing structure, and reducing the effective load-bearing area of the sample. As the effective carrying area decreases, the corresponding values of σ and E show a decreasing trend.

### 3.2. Macroscopic failure characteristics

**Fig 5:**
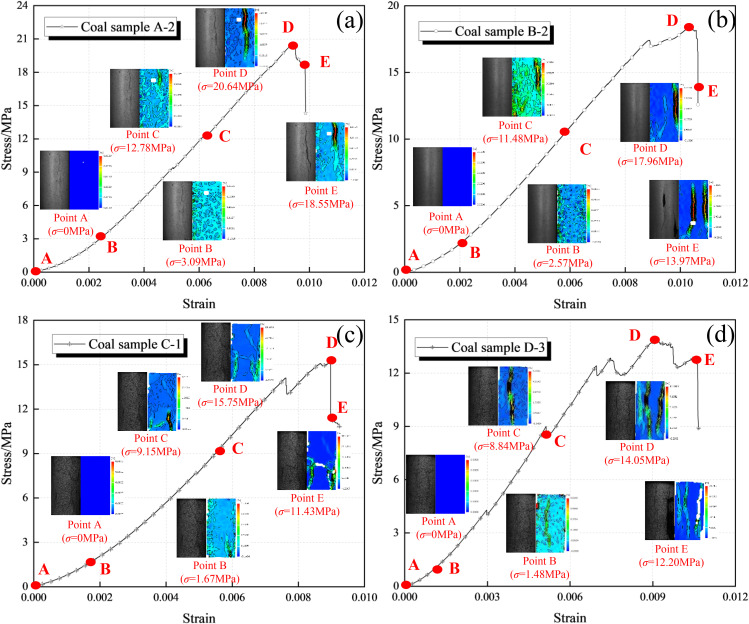
Macroscopic failure characteristics of coal samples. (a)Coal sample A-2. (b)Coal sample B-2. (c)Coal sample C-1. (d)Coal sample D-3.

In order to further study the evolution law of macroscopic crack extension of coal samples, coal samples A-2, B-2, C-1 and D-3 were selected to analyse the characteristics of the deformation field evolution during the uniaxial compression test, and in order to facilitate the subsequent analysis [53,54], five characteristic points (A ~ E) on the stress-strain curves of the coal samples were finally selected to analyse the evolution law of the maximum principal strain field corresponding to the time when the deformation field started to evolve in the first place (Fig 5). The maximum principal strain field evolution pattern is analysed.

As can be seen from Fig 5, at the early stage of loading (characteristic point B), all groups of coal samples are in the stage of compression density, their internal microcracks, pores and other defects are compressed and compacted, the maximum principal strain field is uniformly distributed, and the displacement misalignment is in the stage of micro-variation; compared with the A-2 coal samples, the strain isotropic lines of the B-2, C-1, and D-3 samples are richly developed, which indicates that with the increase of the immersion pressure, the more developed the coal samples are with internal microcracks, pores and other defects. The maximum principal strain field is relatively active at the beginning of loading. Under the action of axial stress, when entering the plastic stage (characteristic point C), the coal samples with larger immersion pressure produce stress concentration at the primary cracks faster, and the strain isopotential lines converge around the primary cracks; take the coal sample D-3 as an example, the deformation localization zone appears first, and the strain isopotential lines of the coal samples converge to the deformation localization zone area, and the development and expansion of the primary macroscopic cracks of the coal samples in this stage is sufficiently extended, and more new cracks are generated. The deformation localisation zones of coal samples C-1 and B-2 enter the growth stage in turn.Compared with the A-2 coal sample, the deformation localization regions of the B-2, C-1, and D-3 coal samples appeared earlier, with larger localized areas and more significant strain contour gradients. This indicates that under axial stress, with the increase of impregnation pressure, stress concentration is more likely to occur at a large number of defects inside the coal sample, leading to the early manifestation of localized deformation areas. When the pressure reaches the critical value required for coal sample instability (characteristic point D), the primary cracks and newly formed cracks in each group of coal samples begin to cross and infiltrate each other, and the stress concentration phenomenon at the macroscopic cracks that control damage is significantly enhanced. The displacement offset in the localized region increases sharply. In the post peak stage (characteristic point E), the coal sample as a whole undergoes damage, sliding along the surface of the main macroscopic crack, and the localized deformation area evolves relatively violently. However, as the impregnation pressure of the coal sample increases, the development of derivative cracks becomes more complete, and the relative displacement between them also increases.

### 3.3. Energy evolution laws

Coal sample destruction is an energy-driven state instability phenomenon from the energy point of view of coal sample instability destruction process analysis, study its uniaxial compression deformation destruction process of the energy evolution law and the relationship between the strength, destruction, can reveal different pressure water immersion under the coal sample mechanical properties of the degradation of the essential features [55–57].

**Fig 6:**
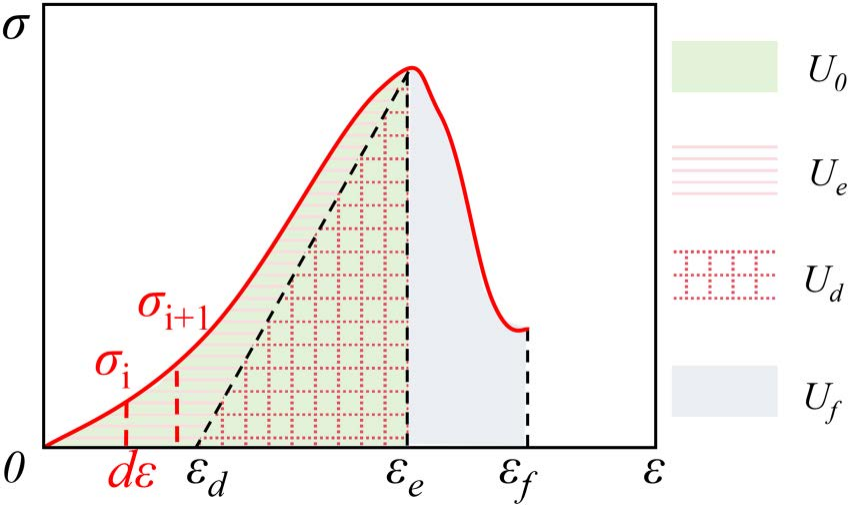
Energy division of coal sample.


*According to the first law of thermodynamics, assuming that there is no heat exchange between the coal sample and the outside world when the external force does work, the flow direction of the external input coal sample energy mainly includes the dissipative energy of the plastic deformation of the coal sample and the development and expansion of holes, cracks, etc., and the elastic energy stored in the solid bearing structure. Fig 6 shows the energy conversion relationship of coal sample unit during uniaxial compression. Dissipative energy density (U*_d_*) can be expressed by total energy density (U*_O_*) and elastic energy density (U*_e_) [58]:


$$U_{o}=U_{e}+U_{d}$$ (1)

According to Fig 6, U_O_ can be expressed as [58]

$$U_{o}=\int_{\text{0}}^{\varepsilon_{\text{c}}}\sigma_{\text{i}}d\varepsilon$$ (2)

Where, σ_i_-stress at any point on the stress-strain, MPa; ε_e_-peak strain.

The coal samples U_e_ and U_d_ were [58]


$$U_{e}=\frac{\text{1}}{\text{2}}\sigma_{\text{c}}\Delta \varepsilon_{\text{e}}=\frac{\text{1}}{\text{2}E}\sigma_{c}^{\text{2}}$$ (3)

$$U_{d}=U_{o}-U_{e}=\int_{\text{0}}^{\varepsilon_{c}}\sigma_{\text{i}}d\varepsilon -\frac{\text{1}}{\text{2}E}\sigma_{c}^{\text{2}}$$ (4)

Where, σ_i_-peak stress, MPa; Δε_e_-recoverable strain; E-unloading modulus of coal samples, MPa, the elastic modulus is taken instead of the unloading modulus in the calculation.

*The post-peak release energy U*_f_ of a coal sample is the area of the stress-strain curve envelope from ε_e_ to ε_f_*, and its magnitude is* [58]:


$$U_{f}=\int_{\varepsilon_{c}}^{\varepsilon_{f}}\sigma_{\text{i}}d\varepsilon$$ (5)

where ε_f_-the maximum strain of the stress-strain curve.

In the post-peak stage, a part of U_e_ is converted into U_f_ and another part into surplus energy U_y_, the magnitude of surplus energy is related to the strength of the dynamic manifestation of the coal sample at the time of destruction, and U_y_ is calculated as [58]


$$U_{y}=U_{e}-U_{f}=\frac{\text{1}}{\text{2}E}\sigma_{c}^{\text{2}}-\int_{\varepsilon_{c}}^{\varepsilon_{f}}d\varepsilon$$ (6)

The value of each energy during uniaxial compression of the coal sample can be calculated from Eqs. (1)~(6) and plotted as shown in Fig 10.

**Fig 7:**
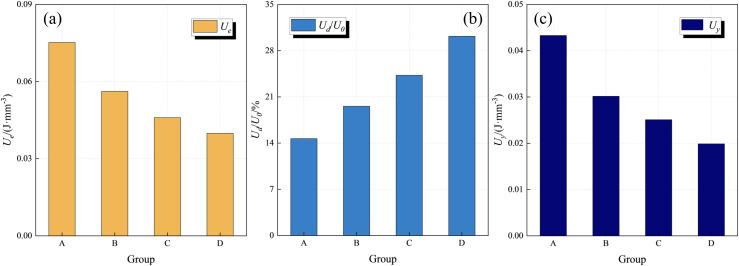
Average energy density value of each group of coal samples. (a) *U*_*e*_. (b) *U*_*d*_*/U*_*0*_. (c)*U*_*y*_.

**Fig 8:**
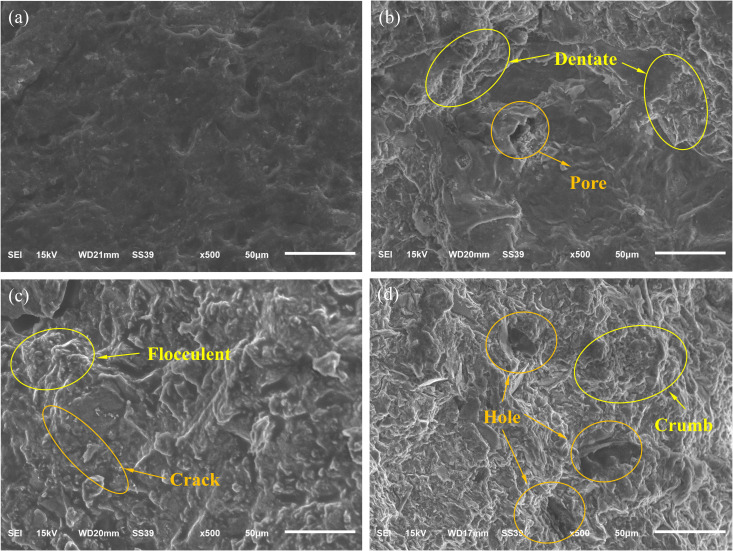
Macroscopic failure characteristics of coal samples. (a) Coal sample A-2. (b) Coal sample B-2. (c) Coal sample C-1. (d) Coal sample D-3.

**Fig 9:**
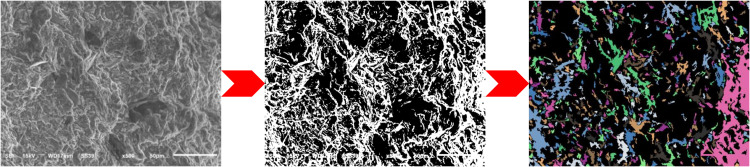
PCAS system process.

**Fig 10:**
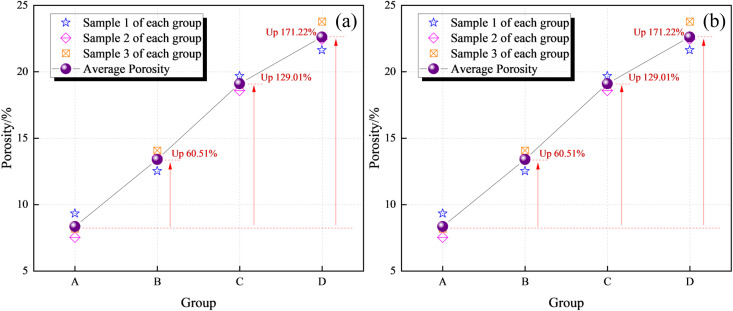
Porosity and probability entropy at fracture surface of coal sample. (a)Porosity. (b)Probability entropy.

Fig 7 shows the comparison of energy density values of coal samples. In the pre-peak stage, the energy input from outside is mainly stored in the form of U_e_ in the solid bearing structure of coal samples, and when Ue reaches the energy storage limit of the bearing structure, the coal samples will be damaged. As shown in Fig 10, with the increase of water immersion pressure, the solid bearing structure of coal samples is weakened, resulting in the decrease of its energy storage limit, and the U_e_ gradually decreases, and compared with the coal samples of group A, the average U_e_ of the coal samples of group B ~ D shows an increasing trend; and the ratio of the corresponding U_d_ to U_0_ shows an increasing trend with the increase of the water immersion pressure. This indicates that under pressure water immersion conditions, the interaction between water and coal is enhanced, and defects such as pores and cracks appear in the coal sample. The damage to the coal sample intensifies, the strength of its solid bearing structure decreases, the energy storage capacity decreases, and the corresponding Ue value gradually decreases; At the same time, the energy input from the outside is used for the plastic deformation of the coal sample during the pre peak stage and the subtle damage caused by the initiation and propagation of microcracks.

In the post-peak stage, with the increase of water immersion, U_y_ shows a decreasing trend, which is due to the fact that under the same conditions, more U_e_ is converted into U_f_, and U_y_ gradually decreases, resulting in a gradual decrease in the power apparent strength of the coal samples at the time of destruction; a large amount of U_e_ is used for the macroscopic cracks within the coal samples to agglomerate through the coal samples, however, with the increase of immersion pressure, the internal cracks continue to expand through the coal samples to mainly occur in the ductile damage, and the coal samples at this time will not immediately lose bearing capacity, but continue to release energy [59–62]. At this time, the coal samples do not lose the bearing capacity immediately, but release energy continuously, and the corresponding stress-curve of the coal samples is more extended in the post-peak stage.

### 3.4. Mesoscopic deterioration characteristics

The rupture fracture morphology can study the internal fine-scale characteristics of coal samples, and the rupture fracture of coal samples under the water pressure of different mine water contains rich information, which is of great significance for us to study the deterioration characteristics from the fine-scale point of view [63–67]. Coal samples A-2, B-2, C-1 and D-3 were selected and their rupture fracture morphology was analysed, as shown in Fig 8.

As can be seen from Fig 8(a),The fracture surface structure of coal sample A-1 is dense, with few cracks and pores, and the surface is smooth and angular. Due to the relative internal integrity of the coal sample, the corresponding σ is larger. With the increase of immersion water pressure, the pressure water-coal action is enhanced, and microcracks and large pores gradually appeared at the fracture of the coal samples, and gradually showed a broken shape; compared with the A-2 coal samples, the rupture fracture of the B-2, C-1 and D-3 coal samples has relatively more microcracks and pores, and the pores are larger, and the particles are more loose, and the structure of the coal samples is changed from dense to loose and porous, see Fig 8(b)~8(d). This indicates that with the prolongation of the pressure water immersion time, which promotes the water-coal interaction, the newborn defects inside the coal samples are loose in structure, and then σ decreases under the action of axial stress.

In order to further quantitatively analyse the fine-scale deterioration characteristics at the rupture fracture of coal samples, PCAS image recognition and analysis system [68,69] was used to quantitatively analyse the pores as well as the fissures of the coal samples under different pressure water immersion. The system imports porous images through binary processing, automatically removes stray waves, automatically segments and identifies pores, outputs geometric and statistical parameters, displays a rose chart, and calculates each parameter of the coal sample SEM image. Fig 9 demonstrates the binarized image of the pore space of the D-1 coal sample processed by PCAS image software, where the black area represents the pore space area and the coloured area represents the non-porous area.

Fig 10 shows the porosity and probability entropy at the rupture fracture of each group of coal samples, and it can be seen from the figure that with the increase of immersion pressure, the porosity and probability entropy of the coal samples are in the increasing trend; the probability entropy is an index describing the directionality of the pores, and the directionality parameter of the microscopic pores in the SEM image can be obtained through the PCAS image analysis software (the probability entropy index ranges from 0 to 1, and all the pore directions in the image are in a random distribution when the value of the probability entropy is 0). consistent, while when the probability entropy value is 1, all pore directions in the image are randomly distributed). The probability entropy is calculated as [70]:


$$H_{m}=-\sum_{i=1}^{n}\frac{m_{i}}{M1}\frac{\ln\left(\frac{m_{i}}{M1}\right)}{\ln n}$$ (7)

where *H*_*m*_ is the probability entropy; *m* is the number of intervals in the i-th interval in the direction of the long axis of the pore; *M* is the total number of particles; *n* is the number of azimuthal zones equally divided in the direction of the alignment of the unit cells (0° to 180°) in units of 10°, i.e., *n* = 18.

This is mainly because with the increase of water immersion pressure, the pressure water coal interaction is enhanced, and the hydrophilic clay minerals in the coal sample can cause the internal holes of the coal sample to expand, and the cracks are easier to expand and penetrate, resulting in the increase of the number of pores at the fracture surface of the coal sample, and the poor orientation of the pores, so the porosity and probability entropy increase.

### 3.5. Mineral composition variation law

**Fig 11:**
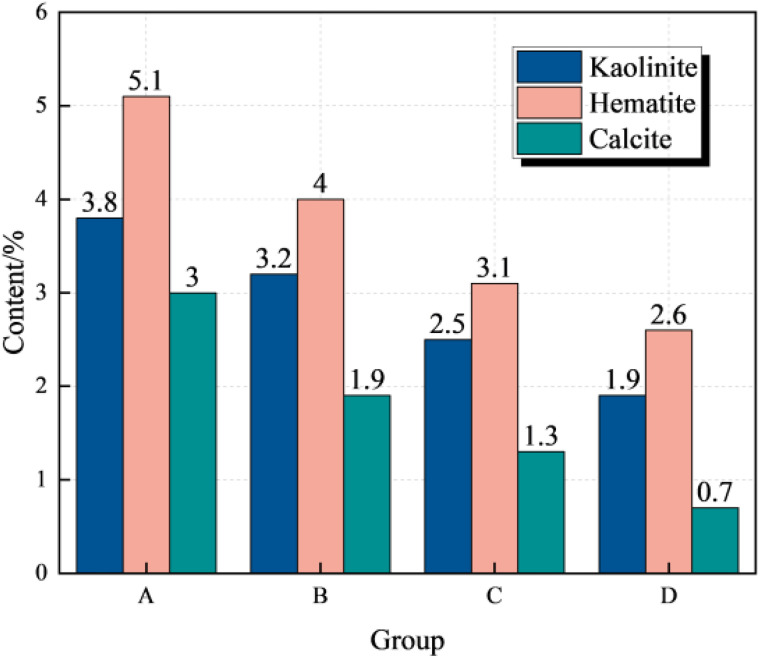
Variation law of mineral composition in coal samples.

In order to study the weakening law of coal and rock by water immersion, it is necessary to further detect the mineral composition of coal samples under pressure water immersion [71–73]. At present, it is generally believed that kaolinite, illite and other clay minerals in the rock have very strong hydrophilicity [74–80], so the content of kaolinite, calcite and hematite in coal samples A-2, B-2, C-1 and D-3 is detected. The test results are shown in Fig 11.

It can be seen from Fig 11 that with the increase of immersion pressure, the mineral contents of Kaolinite, calcite and hematite in group a ~ d coal samples show a decreasing trend. Compared with group a coal samples, the contents of Kaolinite and calcite in group D coal samples are reduced by 50.0% and 76.7% respectively. This is mainly due to the fact that when the coal sample is first soaked, the mine water is acidic, the aluminosilicate minerals and hydrophilic clay minerals (Illite, Kaolinite, etc.) in the coal sample react with H^+^ in the solution rapidly or react with water molecules in the soaking solution through ion exchange or hydrolysis, and some clay mineral components (Illite, Kaolinite, Hematite) soften, argillate, and dissolve in the mine water solution when meeting the mine water, resulting in an increase in pH value, resulting in an increase in the content of Ca^2+^, Al^3+^ and Fe^3+^ in the coal sample soaking solution, resulting in a decrease in the proportion of minerals after soaking.

Reaction process of Calcite and H^+^ ion:


$$CaCO_{\text{3}}+\text{2}H^{+}=Ca^{\text{2}+}+H_{\text{2}}O+CO_{\text{2}}$$ (8)

Reaction process of Kaolinite and H^+^ ion:


$$A{\text{l}}_{\text{2}}Si_{\text{2}}O_{\text{5}}{\left(OH\right)}_{\text{4}}+\text{6}H^{+}=\text{2}Al^{\text{3}+}+\text{2}SiO_{\text{2}}+\text{5}H_{\text{2}}O$$ (9)

Reaction process of Hematite and H^+^ ion:


$$Fe_{\text{2}}O_{\text{3}}+\text{6}H^{+}=\text{2}Fe^{\text{3}+}+\text{3}H_{\text{2}}O$$ (10)

## 4. Discussion

**Fig 12:**
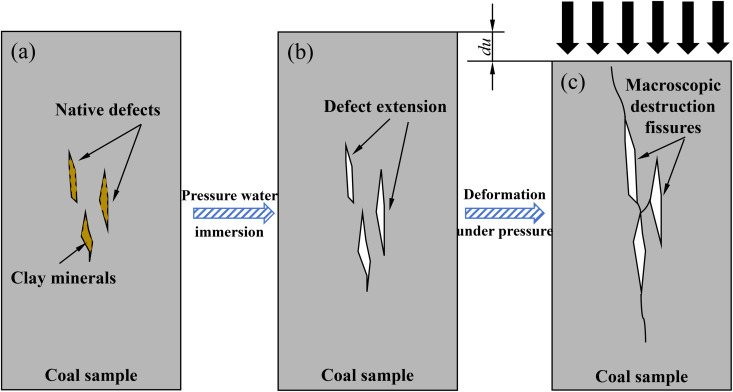
Schematic diagram of damage evolution process of coal sample. (a)Initial state. (b)Pressure water immersion. (c)Deformation under pressure.

As a special rock with discontinuous homogeneity and anisotropy, coal is loose and soft, and defects such as primary fissures and pores are developed. In order to further explore the macroscopic and microscopic deterioration mechanism of the mechanical properties of coal under mine water immersion conditions, a schematic diagram of the damage evolution process of coal under the action of mine water immersion was drawn as shown in Fig 12.

From Fig 12, it can be seen that under the dry condition, the coal samples have good integrity, the structure of primary macroscopic cracks, microcracks and pores is stable and less, the coal particles are mainly linked by cement, the internal defects are uniformly distributed, and they are filled by clay minerals, and the coal samples have a dense structure; after coal samples are subjected to the action of pressure water immersion, the water molecules enter into the interior of the samples through pore cracks and are adsorbed on the clay minerals and the cement, which promotes the pressure-water- Coal-rock action, resulting in the expansion of macro cracks, micro cracks, pore, pore part of the hydrophilic clay minerals and cement softening, sludge and dissolution, coal samples internal pore hollowing to form damage, pore, pore expansion; in the axial stress, due to the pressure of water-coal-rock action caused by the internal defects of coal samples intensified, resulting in the coal samples of the smaller area of the effective load-bearing structure, which led to the weakening of the load-bearing structure of the body, and finally The corresponding uniaxial compressive strength and modulus of elasticity of the coal samples are reduced, and at the same time, due to the formation of pore water pressure by the undischarged water at the internal defects of the coal samples, which leads to the enhancement of the driving force for the emergence and expansion of the macroscopic cracks, microcracks and pores in this area, the degree of fragmentation of coal samples is intensified by the action of the axial stress, and deformation and damage are more prone to occur.

This paper carries out a series of researches on the water pressure effect and influence mechanism of the mechanical properties of coal samples under the conditions of mine water immersion, although some useful research results have been obtained, but water immersion of coal is a complex process, there are still many problems that need to be more in-depth research and discussion:

(1) The immersion test of coal samples carried out in this paper does not take into account the role of overburden loading, and it can not be completely realistic simulation of the site of the complex engineering conditions, the future test can be designed close to the actual site conditions to more comprehensively reveal the influence of the law. In the future, the test can be designed close to the actual field conditions of the simulation test, and then more comprehensively reveal its influence on the law.
(2) The influence of mine water immersion on the strength of coal is a repeated and complex process, while the water pressure is a dynamic change, and this paper only adopts a specific pressure value when researching the influence of mine water on the strength of coal samples, and in the future work, we can consider a longer time and more detailed test programme to meet the actual engineering conditions.

## 5. Conclusions

(1) With the increase of water immersion pressure, the stress-strain curve of coal samples prolongs the compression-density stage, shortens the linear elasticity stage, and the plastic yield stage is relatively short, and the post-peak stage of the coal samples is gradually transformed from brittle to ductile destructive characteristics, and the average uniaxial compressive strength and modulus of elasticity of the samples show a tendency of decreasing.
(2) At the early stage of loading, the distribution of the maximum principal strain field of the coal samples was almost irregular, and with the increase of immersion pressure, the strain isopotential lines of the coal samples were gradually dense; under the action of axial stress, the coal samples firstly produced the stress concentration at the primary macroscopic cracks, formed the deformation localisation zone, and then generated the macroscopic master cracks, and the degree of the coal sample fragmentation was intensified.
(3) During the destruction process, as the immersion pressure increases, the elastic energy stored in the solid load-bearing structure of the coal sample gradually decreases, and its energy storage limit also decreases accordingly. At this point, more external input energy is used for macroscopic and microscopic damage within the plastic range of the coal sample, while the released energy is more used for the aggregation of macroscopic cracks in the coal sample, ultimately leading to a decrease in the dynamic apparent strength of the coal sample.
(4) With the increase of soaking pressure, more microcracks and holes gradually appeared on the rupture fracture surface of coal samples, and the particles were looser from each other, and the structure of coal samples shifted from dense to loose and porous, which resulted in the increase of porosity and probability entropy of rupture fracture of coal samples, and the orientation of pores became poor.
(5) With the increase of soaking pressure, the hydrophilic clay minerals, such as kaolinite, calcite and hematite, etc., in the coal samples had a chemical reaction with the mine water or had an ion exchange or hydrolysis reaction with the water molecules in the coal samples, resulting in a decrease in their content, which verified the law that the pressure water-coal interaction increased with the increase of water pressure.

## Supporting information

S1 Fig (XLSX)

S2 Fig (XLSX)

S7 Fig (XLSX)

S11 Fig (XLSX)

S4A Fig (XLSX)

S4B Fig (XLSX)

S4C Fig (XLSX)

S10A Fig (XLSX)

S10B Fig (XLSX)

## References

[1] Hou Z-M, Xiong Y, Luo J-S, Fang Y-L, Haris M, Chen Q-J, et al. International experience of carbon neutrality and prospects of key technologies: Lessons for China. Petroleum Science. 2023;20(2):893–909. doi: 10.1016/j.petsci.2023.02.018

[2] Xiaocun C, Hui T. Technical scheme and application of pressure-relief gas extraction in multi-coal seam mining region. International Journal of Mining Science and Technology. 2018;28(3):483–9. doi: 10.1016/j.ijmst.2018.03.010

[3] Zhou Y, Sheng Q, Fu X, Ding H. The dynamic deformation properties of rock materials under different types of seismic load. Rock Mech Rock Eng. 2022;55(9):5807–20. doi: 10.1007/s00603-022-02947-z

[4] Maillot A-S, Meyer T, Prunier-Poulmaire S, Vayre E. A qualitative and longitudinal study on the impact of telework in times of COVID-19. Sustainability. 2022;14(14):8731. doi: 10.3390/su14148731

[5] Zhong C, Zhang Z, Ranjith PG, Lu Y, Choi X. The role of pore water plays in coal under uniaxial cyclic loading. Engineering Geology. 2019;257:105125. doi: 10.1016/j.enggeo.2019.05.002

[6] Zhang JC, Lv DW, Zhang JJ, Zhang XY, Yu HY, Li XL, et al. Microfluidic diagnostics: Evolution of gas critical paths based on dynamic alterations of cleats wettability. Chem Eng J. 2025;515(1385-8947):163662. 10.1016/j.cej.2025.163662

[7] Zhang JC, Lv DW, Zhang JJ, Wang F, Yin DW, Yu HY. Dual-scale insights of two-phase flow in inter-cleats based on microfluidics: Interface jumps and energy dissipation. Int J Min Sci Technol. 2025;35(3):451–65. 10.1016/j.ijmst.2025.01.010

[8] Mo P, Tang Q, Luo J, Huang H, Yang Q, Chen Y. Study on time-effect to the strength deterioration of carbonaceous mudstone under water-rock interaction. Matéria (Rio J). 2023;28(3). doi: 10.1590/1517-7076-rmat-2023-0166

[9] Liu S, Zhang K, Gao J, Yang Y, Bai L, Yan J. Research on temporal patterns of water-rock interaction in the coal mine underground reservoir based on the dynamic simulation test. ACS Omega. 2023;8(15):13819–32. doi: 10.1021/acsomega.2c08145 37091424 PMC10116550

[10] Pang J, Wu T, Yu X, Zhou C, Chen H, Gao J. The effect of water–rock interaction on shale reservoir damage and pore expansion. Processes. 2025;13(5):1265. doi: 10.3390/pr13051265

[11] Kang H, Gao F, Xu G, Ren H. Mechanical behaviors of coal measures and ground control technologies for China’s deep coal mines – A review. Journal of Rock Mechanics and Geotechnical Engineering. 2023;15(1):37–65. doi: 10.1016/j.jrmge.2022.11.004

[12] Liu W, Yang K, Zhang S, Zhang Z, Xu R. Energy evolution and water immersion-induced weakening in sandstone roof of coal mines. Int J Coal Sci Technol. 2022;9(1). doi: 10.1007/s40789-022-00529-6

[13] Li FX, Li Z, Xue QQ, Wang S. Failure mechanism of rock-like specimens under uniaxial compression: Effects of hole-crack spatial relationship and crack number. Theor Appl Fract Mech. 2025;140(0167-8442):105185. 10.1016/j.tafmec.2025.105185

[14] Niu S, Ge S, Yang D, Dang Y, Yu J, Zhang S. Mechanical properties and energy mechanism of saturated sandstones. J Cent South Univ. 2018;25(6):1447–63. doi: 10.1007/s11771-018-3839-z

[15] Pu H, Yi Q, Jivkov AP, Bian Z, Chen W, Wu J. Effect of dry-wet cycles on dynamic properties and microstructures of sandstone: Experiments and modelling. International Journal of Mining Science and Technology. 2024;34(5):655–79. doi: 10.1016/j.ijmst.2024.04.008

[16] Zhang Z, Xie H, Zhang R, Zhang Z, Gao M, Jia Z, et al. Deformation damage and energy evolution characteristics of coal at different depths. Rock Mech Rock Eng. 2018;52(5):1491–503. doi: 10.1007/s00603-018-1555-5

[17] Li X, Peng K, Peng J, Xu H. Effect of cyclic wetting–drying treatment on strength and failure behavior of two quartz-rich sandstones under direct shear. Rock Mech Rock Eng. 2021;54(11):5953–60. doi: 10.1007/s00603-021-02583-z

[18] Sun Q, Zhang H, Hu J, Geng J, Zhou S. Damage mechanism of granite under subcritical water–rock interaction. Environ Earth Sci. 2023;82(5). doi: 10.1007/s12665-023-10827-0

[19] Song C, Feng G, Bai J, Cui J, Wang K, Shi X, et al. Progressive failure characteristics and water-induced deterioration mechanism of fissured sandstone under water–rock interaction. Theoretical and Applied Fracture Mechanics. 2023;128:104151. doi: 10.1016/j.tafmec.2023.104151

[20] Zhou KY, Dou LM, Gong SY, et al. Mechanical behavior of sandstones under water-rock interactions. Geomechanics and Engineering. 2022;29(6). doi: 10.12989/gae.2022.29.6.627

[21] Ji J, Zhao J, Yi J, Song X, Wang G. Optimization of hot dry rock heat extraction performance considering the interaction of multi-mineral component water-rock reactions and fracture roughness. Energy. 2025;320:135089. doi: 10.1016/j.energy.2025.135089

[22] Hua W, Li J, Dong S, Pan X. Experimental study on mixed mode fracture behavior of sandstone under water–rock interactions. Processes. 2019;7(2):70. doi: 10.3390/pr7020070

[23] Deng HF, Zhou ML, Li JL, Sun XS, Huang YL. Creep degradation mechanism by water-rock interaction in the red-layer soft rock. Arab J Geosci. 2016;9(12). doi: 10.1007/s12517-016-2604-6

[24] Zhou M, Li J, Luo Z, Sun J, Xu F, Jiang Q, et al. Impact of water-rock interaction on the pore structures of red-bed soft rock. Sci Rep. 2021;11(1):7398. doi: 10.1038/s41598-021-86815-w 33795793 PMC8016936

[25] Liu Y, Wang H, Niu H, Xing S, Wang G, Zhou Z, et al. Coal pore structure evolution under drying–wetting cycle. Nat Resour Res. 2025;34(4):2239–59. doi: 10.1007/s11053-025-10481-2

[26] Liu Z, Wang G, Li J, Li H, Zhao H, Shi H, et al. Water-immersion softening mechanism of coal rock mass based on split Hopkinson pressure bar experiment. Int J Coal Sci Technol. 2022;9(1). doi: 10.1007/s40789-022-00532-x

[27] Yao Q, Chen T, Tang C, Sedighi M, Wang S, Huang Q. Influence of moisture on crack propagation in coal and its failure modes. Engineering Geology. 2019;258:105156. doi: 10.1016/j.enggeo.2019.105156

[28] Yao QL, Wang WN, Li XH, et al, Study of mechanical properties and acoustic emissioncharacteristics of coal measures under water-rock interaction. Journal of China University of Mining &Technology. 2021;50(03):558–69.

[29] Yao QL, Hao Q, Chen XY, et al. Design on the width of coal pillar dam in coal mine groundwater reservoir. Journal of China Coal Society. 2019;44(3):890–8.

[30] Lai XP, Zhang S, Dai JJ, et al. Multi-scale damage evolution characteristics of coal and rock under hydraulic coupling. Chinese Journal of Rock Mechanics and Engineering. 2020;39(S2):3217–28.

[31] Lai XP, Zhang S, Cui F, et al. Energy release law during the damage evolution of water-bearing coal and rock and pick-up of AE signals of key pregnancy disasters. Chinese Journal of Rock Mechanics and Engineering. 2020;39(03): 433–44.

[32] Wang FT, Zhang C, Tang TK, et al. Pore and strength damage evolution mechanism of coal induced by the circulating water immersion effect. Journal of Mining Science and Technology. 2024;9(4), 608–18.

[33] Han PH, Zhao YX, Gao S, et al. Progressive damage characteristics and damage constitutive model of coal samples under long-term immersion. Chinese Journal of Rock Mechanics and Engineering. 2024;43(04):918–33.

[34] Jin F, Wang S, Duan H, Xia Y, Yang T, Yang D. Study on the water absorption characteristics of anthracite particles after immersion in water. ACS Omega. 2024;9(26):28841–51. doi: 10.1021/acsomega.4c03339 38973869 PMC11223212

[35] Lyu K, Jiang N, Yin D, Meng S, Gao Z, Lyu T. Deterioration of compressive properties of coal rocks under water and gas coupling. J Cent South Univ. 2024;31(2):477–95. doi: 10.1007/s11771-024-5583-x

[36] Zhang JC, Lv DW, Yin DW, Zhang XY, Li XL, Fan KK. Gas recovery and flowback in trans-coal-limestone fracture: An in-situ wettability microscale visualization insight. Gas Sci Eng. 2025;142(2949-9089):205707. 10.1016/j.jgsce.2025.205707

[37] General Coal Research Institute GB/t 23561 determination of physical and mechanical properties of coal and rock [s]. Beijing: China Standards Press; 2009.

[38] Zhang Y, Deng H, Wang W, Duan L, Zhi Y, Li J. The dynamic response law of bank slope under water‐rock interaction. Advances in Civil Engineering. 2018;2018(1). doi: 10.1155/2018/1306575

[39] Zhang K, Deng X, Gao J, Liu S, Wang F, Han J. Insight into the process and mechanism of water-rock interaction in underground coal mine reservoirs based on indoor static simulation experiments. ACS Omega. 2022;7(41):36387–402. doi: 10.1021/acsomega.2c04161 36278070 PMC9583642

[40] Luo Z, Li J, Jiang Q, Zhang Y, Huang Y, Assefa E, et al. Effect of the Water-Rock Interaction on the Creep Mechanical Properties of the Sandstone Rock. Period Polytech Civil Eng. 2018. doi: 10.3311/ppci.11788

[41] Zhang H, Liu XL, Huang Z, Lu XL. Study on rock energy evolution and constitutive model under water–rock interaction. IOP Conf Ser: Earth Environ Sci. 2024;1331(1):012003. doi: 10.1088/1755-1315/1331/1/012003

[42] Yang Z, Li S, Liu Y, Zhang N. Experimental study on the influence of water-rock interaction on the time-varying characteristics of coal rock mass. Environ Geochem Health. 2024;46(2):45. doi: 10.1007/s10653-023-01832-0 38227264

[43] Wang B, Gao B, Wang L, Wang S, Wu C, Zhang W, et al. Pore Size Evolutionary Mechanism of Anthracite Induced from Water-Rock Interactions. ACS Omega. 2025;10(8):7635–47. doi: 10.1021/acsomega.4c06280 40060801 PMC11886642

[44] Liu JF, Ding YS, Xue FJ, Wei JB, Lin H, Dai HY. Confinement pressure effect and influence mechanism of water injection-induced slip of shale fracture. Eng Geol. 2025;352(0013-7952):108061. 10.1016/j.enggeo.2025.108061

[45] Yang F, Wang G, Hu D, Zhou H, Tan X. Influence of water-rock interaction on permeability and heat conductivity of granite under high temperature and pressure conditions. Geothermics. 2022;100:102347. doi: 10.1016/j.geothermics.2022.102347

[46] Zhang L, Zhang T, Zhao Y, Hu H, Wen S, Wu J, et al. A review of interaction mechanisms and microscopic simulation methods for CO2-water-rock system. Petroleum Exploration and Development. 2024;51(1):223–38. doi: 10.1016/s1876-3804(24)60019-4

[47] Zhang L, Zhang Q, Zhang X, Zhang J, An Q. Kinetics mechanism of pore pressure effect on CO2-water-rock interactions: An experimental study. Chemical Engineering Journal. 2024;489:151361. doi: 10.1016/j.cej.2024.151361

[48] Zheng P, Tan X, Xie Y, Shen K, Du Z, Zhou Y, et al. Study on the Tensile Strength and Fracture Characteristics of Interlayer Mineral Grain Interfaces in Slate Exposed to Water–Rock Interaction. Rock Mech Rock Eng. 2025;58(9):9801–24. doi: 10.1007/s00603-025-04660-z

[49] Chen Y, Cao P, Mao D, Pu C, Fan X. Morphological analysis of sheared rock with water–rock interaction effect. International Journal of Rock Mechanics and Mining Sciences. 2014;70:264–72. doi: 10.1016/j.ijrmms.2014.05.002

[50] Gao M, Gao Z, Yang B, Xie J, Wang M, Hao H, et al. Macroscopic and microscopic mechanical behavior and seepage characteristics of coal under hydro-mechanical coupling. J Cent South Univ. 2024;31(8):2765–79. doi: 10.1007/s11771-024-5726-0

[51] Zheng Z, Su H, Wang W, et al. A new hydro-mechanical coupling constitutive model for brittle rocks considering initial compaction, hardening and softening behaviors under complex stress states. Geomechanics and Geo-engineering and Geo-energy and Geo-resources. 2023;9:68.

[52] Chen W, Liu B, Wu Q, Liu J, Ren Z, Wang Q, et al. Experimental study on effective stress coefficient of sandstone based on Mohr-Coulomb criterion under hydraulic-mechanical coupling. Sci Rep. 2025;15(1):17437. doi: 10.1038/s41598-025-01976-2 40394174 PMC12092590

[53] Su G, Huang J, Liu Y. Crack propagation mechanism of fissured sandstone subjected to uniaxial cyclic compression test. Theoretical and Applied Fracture Mechanics. 2025;139:105049. doi: 10.1016/j.tafmec.2025.105049

[54] Yin DW, Sun PX, Ding YS, Tan Y, Zhang JC, Li FX. Mechanical properties of pressure water-immersed coal under different cyclic stress paths. Rock Mech Rock Eng. 2025;58(11):11677–95. 10.1007/s00603-025-04713-3

[55] Zhang ZZ, Gao F. Experimental research on energy evolution of red sandstone samples under uniaxial compression. Chinese Journal of Rock Mechanics and Engineering. 2012;31(05):953–62.

[56] Xie HP, Peng RD, Ju Y,. Energy dissipation of rock deformation and fracture. Chinese Journal of Rock Mechanics and Engineering. 2004;(21):3565–70.

[57] Li X, Yao Z, Huang X, Liu X, Fang Y, Xu Y. Mechanical Properties and Energy Evolution of Fractured Sandstone under Cyclic Loading. Materials (Basel). 2022;15(17):6116. doi: 10.3390/ma15176116 36079497 PMC9458138

[58] Xie HP, Ju Y, Li LY. Criteria for strength and structural failure of rocksbased on energy dissipation and energy release principles. Chinese Journal of Rock Mechanics and Engineering. , 2005;(17): 3003–10.

[59] Wang H, Wu Y, Qu X, Wang W, Liu Y, Xie W-C. Experimental and numerical investigations on failure process and permeability evolution of weathered granite under hydro-mechanical coupling. International Journal of Rock Mechanics and Mining Sciences. 2025;191:106117. doi: 10.1016/j.ijrmms.2025.106117

[60] Song J-F, Lu C-P, Zhan Z-W, Cui H-F, Wang Y-M, Wang J-H. Numerical and field investigations of acoustic emission laws of coal fracture under hydro-mechanical coupling loading. Materials (Basel). 2022;15(19):6510. doi: 10.3390/ma15196510 36233850 PMC9572184

[61] Galav A, Singh GSP, Sharma SK. Hydro-mechanically coupled numerical modelling of protective water barrier pillars in underground coal mines in India. Mine Water Environ. 2023;42(3):418–40. doi: 10.1007/s10230-023-00946-2

[62] Tan T, Zhang C, Zhao Y, Li X. Mechanical behavior and damage constitutive model of sandstone under hydro-mechanical (H-M) coupling. International Journal of Mining Science and Technology. 2024;34(6):837–53. doi: 10.1016/j.ijmst.2024.07.002

[63] Sun X, Zhang J, Shi F, He L, Zhang Y, Miao C, et al. Failure microscopic mechanism and damage constitutive model of dolomite under water-rock coupling interaction. J Cent South Univ. 2025;32(4):1431–46. doi: 10.1007/s11771-025-5939-x

[64] Wan F, Zhang H, Zhou P, Guo J. Determination of water‐proof coal (rock) pillar height in mining coal seam group under water‐bearing rock stratum. Shock and Vibration. 2021;2021(1). doi: 10.1155/2021/6699726

[65] Wu C, Zhu H, Lu Y, Zhao D, Chu J, Qi Y. Comparative evaluation of the effects of long-term and short-term water–rock interaction on the microstructure and mechanical properties of shale. Energy Fuels. 2025;39(7):3540–63. doi: 10.1021/acs.energyfuels.4c06122

[66] Lee SG, Kim JC. Preliminary experimental result for clarifying sr isotope behaviour of water due to water-rock interaction. Economic and Environmental Geology. 2010;43(3).

[67] Wang F, Cao P, Cao R, Xiong X, Hao J. The influence of temperature and time on water-rock interactions based on the morphology of rock joint surfaces. Bull Eng Geol Environ. 2018;78(5):3385–94. doi: 10.1007/s10064-018-1315-5

[68] Liu C, Shi B, Zhou J, Tang C. Quantification and characterization of microporosity by image processing, geometric measurement and statistical methods: Application on SEM images of clay materials. Applied Clay Science. 2011;54(1):97–106. doi: 10.1016/j.clay.2011.07.022

[69] Liu C, Xu Q, Shi B, et al. Digital image recognition method of rock particle and pore system andits application. Chinese Journal of Geotechnical Engineering. 2018;40(05):925–31.

[70] Shi B. Quantitative assessment of changes of microstructure for clayey soil in the process of compaction. Chinese Journal of Geotechnical Engineering. 1996;(02):60–5.

[71] Mo P, Tang QZ, Luo JH, et al. Study on time-effect to the strength deterioration of carbonaceous mudstone under water-rock interaction. 2023;28(03).

[72] Liu SY, Zhang K, Gao J, et al. Research on temporal patterns of water-rock interaction in the coal mine underground reservoir based on the dynamic simulation test. 2023;08(15):13819–32. 10.1021/acsomega.2c08145 PMC10116550 37091424

[73] Pang J, Wu TT, Yu XN, et al. The Effect of Water-Rock Interaction on Shale Reservoir Damage and Pore Expansion. 2025;13(05).

[74] Xu J, Sun H, Cui Y, Fei D, Lan H, Yan C, et al. Study on dynamic characteristics of diorite under Dry–wet cycle. Rock Mech Rock Eng. 2021;54(12):6339–49. doi: 10.1007/s00603-021-02593-x

[75] Li R, Zhou C, Fu Y, Zhu J. Assessments of tensile characteristics and degradation mechanism of sandstone subjected to wetting–drying cyclic treatment. Rock Mech Rock Eng. 2023;56(12):8965–78. doi: 10.1007/s00603-023-03539-1

[76] Wang H, Bi J, Zhao Y, Wang C, Ma J. NMR-based analysis of the effect of moisture migration on sandstone pore structure under alternating wetting and drying conditions. International Journal of Mining Science and Technology. 2024;34(8):1135–50. doi: 10.1016/j.ijmst.2024.07.014

[77] Zhang C, Bai Q, Han P. A review of water rock interaction in underground coal mining: problems and analysis. Bull Eng Geol Environ. 2023;82(5). doi: 10.1007/s10064-023-03142-2

[78] Apollaro C, Perri F, Borrelli L, Caloiero T. The role of water-rock interaction processes in soil formation: geochemical, mineralogical, geomorphological, and engineering-geological aspects. Geofluids. 2019:1–4. doi: 10.1155/2019/8453136

[79] Yang X, Wang J, Hou D, Zhu C, He M. Effect of dry-wet cycling on the mechanical properties of rocks: a laboratory-scale experimental study. Processes. 2018;6(10):199. doi: 10.3390/pr6100199

[80] Knuth C, Wohnlich S. Temperatur- und Druckabhängigkeit von Wasser-Gesteins-Wechselwirkungen unter Berücksichtigung der Reaktionskinetik. Grundwasser - Zeitschrift der Fachsektion Hydrogeologie. 2020;25(3):205–13. doi: 10.1007/s00767-020-00456-w

